# Designing Ambient Pressure Superconductivity in Li–Mg-Based Hydrides via Transition-Metal Modulation: From Quaternary to Quinary Systems

**DOI:** 10.3390/ma19163465

**Published:** 2026-08-16

**Authors:** Xinyu Wang, Qun Wei, Jing Luo, Meiguang Zhang

**Affiliations:** 1 School of Physics, Xidian University, Xi’an 710071, China; 2 College of Physics and Optoelectronic Technology, Baoji University of Arts and Sciences, Baoji 721016, China

**Keywords:** superconductivity, multicomponent hydrides, electronic characteristics, first-principles calculations

## Abstract

Hydrogen-rich superconductors have emerged as promising candidates for achieving high-temperature superconductivity, yet their practical applications are generally limited by the requirement for high external pressures. In this work, taking the *Fm*-3-XYZ_2_H_12_ structure as a prototype, we constructed a series of LiMgM_2_H_12_ quaternary hydrides and the LiMgZrHfH_12_ quinary hydride. High-throughput screening indicates that the three hydrides, LiMgZr_2_H_12_, LiMgHf_2_H_12_, and LiMgZrHfH_12_, are dynamically stable at ambient pressure but thermodynamically metastable. Electron–phonon coupling calculations show that the *T*_c_ values of LiMgZr_2_H_12_, LiMgHf_2_H_12_, and LiMgZrHfH_12_ reach 87.4, 81.2, and 85.2 K at ambient pressure, respectively, all exceeding the boiling point of liquid nitrogen and demonstrating excellent superconducting properties. Further analysis reveals that, compared with the Ga-based parent *Fm*-3-XYZ_2_H_12_ hydrides, Li substitution markedly reconstructs the electronic-state distribution near the Fermi level and enhances the contribution of H atoms to the density of states near the Fermi level. This promotes the coupling between conducting electrons and high-frequency hydrogen vibrations. Such strong electron–phonon coupling plays a crucial role in their high-temperature superconductivity. These findings provide valuable insights into the theoretical design of high-*T*_c_ superconductors under ambient pressure and offer theoretical guidance for future experimental studies in this field.

## 1. Introduction

Superconductors, owing to their unique physical properties, including zero-resistance electrical transport and perfect diamagnetism, are widely regarded as ideal materials with tremendous potential for applications in low-loss power transmission, high-field magnetic devices, magnetic levitation, and quantum computing. Consequently, the exploration of superconducting materials has remained one of the most challenging frontiers in condensed matter physics and materials science. Since the establishment of the Bardeen–Cooper–Schrieffer (BCS) theory [1], metallic hydrogen has been predicted to be one of the ideal candidate systems for achieving a high superconducting transition temperature (*T*_c_) because of its high-frequency phonon characteristics and strong electron–phonon coupling (EPC) [2]. However, the realization of metallic hydrogen requires extremely high pressures exceeding 400 GPa, making its experimental synthesis nearly unattainable [3,4,5].

To overcome this challenge, Ashcroft proposed the concept of chemical precompression [6], in which metal elements are introduced into the hydrogen lattice to reduce the pressure required for metallization through the internal compressive effect exerted by metal atoms on the hydrogen sublattice. The successful implementation of this strategy has led to the discovery of a series of high-temperature superconducting hydrides, including the binary covalent hydride H_3_S (203 K at 155 GPa) [7,8], the clathrate superhydrides LaH_10_ (250–260 K at 170–188 GPa) [9,10], YH_6_ (224 K at 166 GPa) [11], YH_9_ (243 K at 201 GPa) [12], and CaH_6_ (215 K at 172 GPa) [13]. These discoveries have convincingly demonstrated the effectiveness of the chemical precompression strategy in promoting high-temperature superconductivity. However, these representative high-*T*_c_ hydrides generally require pressures on the order of hundreds of GPa or even higher to maintain structural stability, which severely limits their synthesizability and potential applications. Therefore, achieving the stabilization of hydrogen-rich compounds at lower pressures, or even under ambient conditions, while preserving excellent superconducting properties has become a critical bottleneck that urgently needs to be addressed in the field of hydrogen-based superconductivity research.

In recent years, the design of multicomponent hydrides has provided a new avenue for reducing the stabilization pressure of hydrides and tuning their superconducting properties. Compared with binary hydrides, ternary, quaternary, and even higher-component hydrides possess richer chemical compositions, local coordination environments, and degrees of freedom in their electronic structures. These features allow the bonding characteristics of the hydrogen sublattice, the density of states near the Fermi level, and phonon softening behavior to be tuned through synergistic interactions among metal elements [14,15,16]. However, for multicomponent hydride systems containing four or more constituent elements, the structural search space expands rapidly as the number of elemental species and possible configurations increases. As a result, conventional crystal structure prediction methods, such as USPEX [17], CALYPSO [18], and AIRSS [19], face substantial computational costs when applied to these multicomponent systems. Recent studies indicate that targeted design using known high-temperature superconducting hydrides as structural templates is an effective strategy for realizing multicomponent hydrides with high *T*_c_ at low pressure. For example, Wei et al. [20] designed a series of quaternary hydrides based on the *Fm*-3*m*-XZH_8_ prototype through substitutional doping. Among them, LaThBe_2_H_16_, AcBaSi_2_H_16_, AcSrSi_2_H_16_, and YceBe_2_H_16_ exhibit *T*_c_ values of 126, 137, 123, and 105 K at 10, 30, 30, and 45 GPa, respectively. Zhao et al. [21] constructed a series of XM_3_Be_4_H_32_ structures based on the LaBeH_8_ prototype, in which CaTh_3_Be_4_H_32_, ThLa_3_Be_4_H_32_, LaAc_3_Be_4_H_32_, and AcLa_3_Be_4_H_32_ achieve *T*_c_ values exceeding 120 K at relatively low pressures. Furthermore, Shi et al. [22] designed the MX_3_B_4_H_20_ family of quaternary hydrogen-rich compounds using the ThBH_5_ parent structure as the structural template. The *T*_c_ values of LaNa_3_B_4_H_20_, ThLa_3_B_4_H_20_, NaTh_3_B_4_H_20_, and LaTh_3_B_4_H_20_ are 143 K (50 GPa), 122 K (40 GPa), 118 K (50 GPa), and 112 K (30 GPa), respectively. These studies demonstrate that quaternary hydrides can effectively reduce the stabilization pressure of hydrides through the synergistic effects of multielement chemical precompression and electronic modulation. Moreover, they substantially expand the materials design space for achieving high-temperature superconductivity at relatively low pressures.

Among hydrogen-rich superconducting systems, A15-type structures have attracted considerable attention because of their highly symmetric framework and the tunable potential for multicomponent alloying [23,24]. Liu et al. [25] proposed a multiregion elemental regulation strategy based on an alloyed A15 framework. By selecting and combining elements from different regions of the periodic table, they constructed a series of quaternary XYZ_2_H_12_ hydrides, among which GaAlZr_2_H_12_ and GaAlHf_2_H_12_ exhibit *T*_c_ values of 74 and 69 K at 20 GPa, respectively. Notably, in our previous work [26], LiMgZr_2_H_12_ was designed through Li substitutional doping based on the MgZrH_6_ parent structure. This compound exhibits a *T*_c_ of 60.8 K at ambient pressure. A comparison of its superconducting properties with those of the parent structure shows that the required external pressure for structural stabilization is significantly reduced while the *T*_c_ is essentially preserved.

Inspired by the above studies, in this work, we constructed a series of LiMgM_2_H_12_ (M = 3*d*, 4*d*, and 5*d* transition metals) quaternary hydrides through elemental substitution based on the *Fm*-3-XYZ_2_H_12_ prototype. We performed high-throughput screening of the stability of the obtained compounds at ambient pressure and systematically evaluated their thermodynamic, mechanical, and dynamical stability. Two dynamically stable compounds at ambient pressure were identified: LiMgZr_2_H_12_ and LiMgHf_2_H_12_. EPC calculations indicate that both compounds are superconductors. LiMgZr_2_H_12_ and LiMgHf_2_H_12_ exhibit *T*_c_ values of 87.4 and 81.2 K at ambient pressure, respectively, both exceeding the liquid-nitrogen temperature and demonstrating excellent superconducting properties. However, existing studies have primarily focused on ternary and quaternary hydride systems, whereas the extension of the elemental substitution strategy to quinary hydrides has rarely been explored. Considering that synergistic effects in multicomponent systems may have an important influence on superconducting properties, the exploration of quinary compounds is of significant scientific importance. Therefore, in this work, we further extend the elemental substitution strategy from quaternary to quinary systems. Based on the LiMgM_2_H_12_ structural prototype, two transition metal elements, Zr and Hf, are simultaneously introduced into the A15 framework to construct the quinary hydrogen-rich compound LiMgZrHfH_12_. Its structural stability, electronic properties, and superconductivity are then systematically investigated. This work advances the study of multicomponent hydride superconducting systems from quaternary to quinary compositions, providing a new research paradigm for compositional design and property regulation of ambient-pressure high-temperature superconducting hydrides. It is of great significance for promoting the development of hydrogen-rich compounds toward practical ambient-pressure superconducting materials.

## 2. Computational Details

Structural optimizations and subsequent property calculations were performed within the framework of density functional theory (DFT) using the Vienna *Ab initio* Simulation Package (VASP) [27]. The exchange–correlation functional was approximated using the Perdew–Burke–Ernzerhof (PBE) functional form within the generalized gradient approximation (GGA) [28], while the ion–electron interactions were described by the projector augmented-wave (PAW) method [29]. Structural relaxations were performed with a 600 eV plane-wave cutoff and a 2π × 0.02 Å^−1^ Monkhorst–Pack *k*-point mesh [30], achieving force convergence below 1 × 10^−3^ eV/Å and total energy convergence within 1 × 10^−5^ eV/atom. The single-crystal elastic constants *C*_ij_ were extracted from the linear stress–strain responses obtained under small finite deformations [31]. The dynamical stability was examined by the finite-displacement approach, with phonon dispersion curves calculated using the PHONOPY package [32]. Electron–phonon coupling (EPC) calculations were carried out within density-functional perturbation theory as implemented in the Quantum ESPRESSO package [33], employing ultrasoft pseudopotentials with a plane-wave kinetic-energy cutoff of 80 Ry. The self-consistent charge density was obtained using a dense 20 × 20 × 20 *k*-point mesh, while the EPC constants were evaluated on a 5 × 5 × 5 *q*-point grid. The superconducting transition temperature *T*_c_ was estimated using the Allen–Dynes modified McMillan formula [34,35]

(1) $$T_{c}=\omega_{\log}\frac{f_{1}f_{2}}{1.2}\exp\left(\frac{-1.04\left(1+\lambda \right)}{\lambda -\mu *-0.62\lambda \mu *}\right)$$

where *f*_1_ and *f*_2_ represent the strong-coupling and shape correction factors, respectively; *ω*_log_ denotes the logarithmic average phonon frequency; *λ* is the dimensionless electron–phonon coupling constant that characterizes the strength of the attractive phonon-mediated interaction; and μ* is the Coulomb pseudopotential describing the effective screened electron–electron repulsion [36,37,38]. The parameter μ* is an empirical parameter that is largely material-independent, with typical values of 0.10–0.13 [39,40,41]. The *ω*_log_ and *λ* were given by:

(2) $$\omega_{\log}=\exp\left(\frac{2}{\lambda }\int \frac{d\omega }{\omega }\alpha^{2}F\left(\omega \right)\ln\left(\omega \right)\right)$$

and

(3) $$\lambda =2\int \frac{\alpha^{2}F\left(\omega \right)}{\omega }d\omega$$

respectively. Accordingly, *λ* is determined from the calculated electron–phonon spectral function and thus directly reflects the electron–phonon coupling strength of the system under consideration. The correction factors *f*_1_ and *f*_2_ are defined as

(4) $$f_{1}=\sqrt[{3}]{1+{\left(\frac{\lambda }{2.46\left(1+3.8\mu *\right)}\right)}^{\frac{3}{2}}}$$

and

(5) $$f_{2}=1+\frac{\left(\frac{{\bar{\omega }}_{2}}{\omega_{\log}}-1\right)\lambda^{2}}{\lambda^{2}+{\left(1.82\left(1+6.3\mu^{*}\right)\frac{{\bar{\omega }}_{2}}{\omega_{\log}}\right)}^{2}}$$

respectively, where ${\bar{\omega }}_{2}$ is the second moment of the normalized weight function, described as

(6) $${\bar{\omega }}_{2}=\sqrt{\frac{2}{\lambda }\int_{0}^{\omega }\alpha^{2}F\left(\omega \right)d\omega }$$

## 3. Results and Discussion

**Figure 1:**
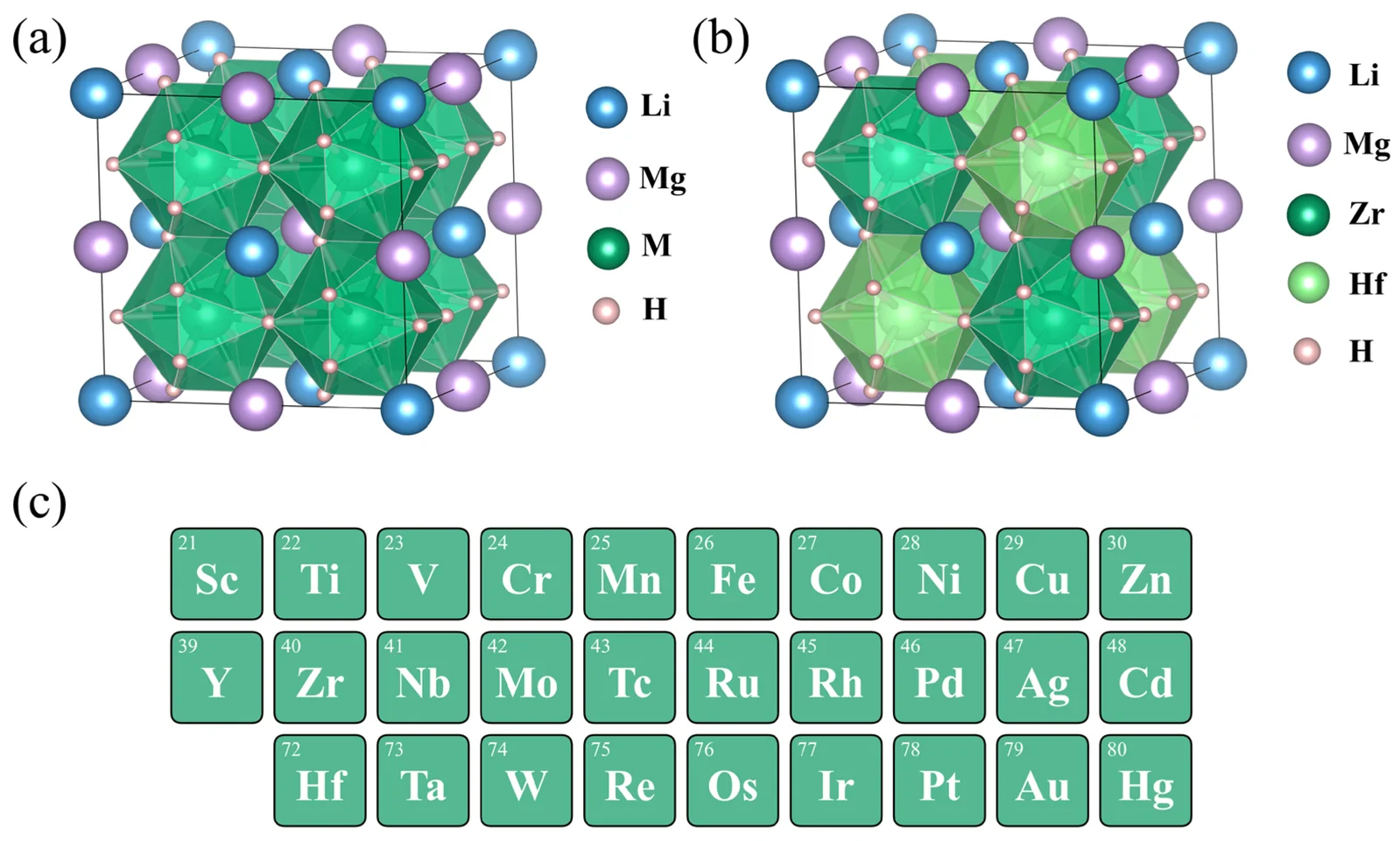
Crystal structure of (**a**) quaternary LiMgM_2_H_12_ and (**b**) quinary LiMgZrHfH_12_ hydrides. (**c**) The selected transition metal range for the M site.

**Figure 2:**
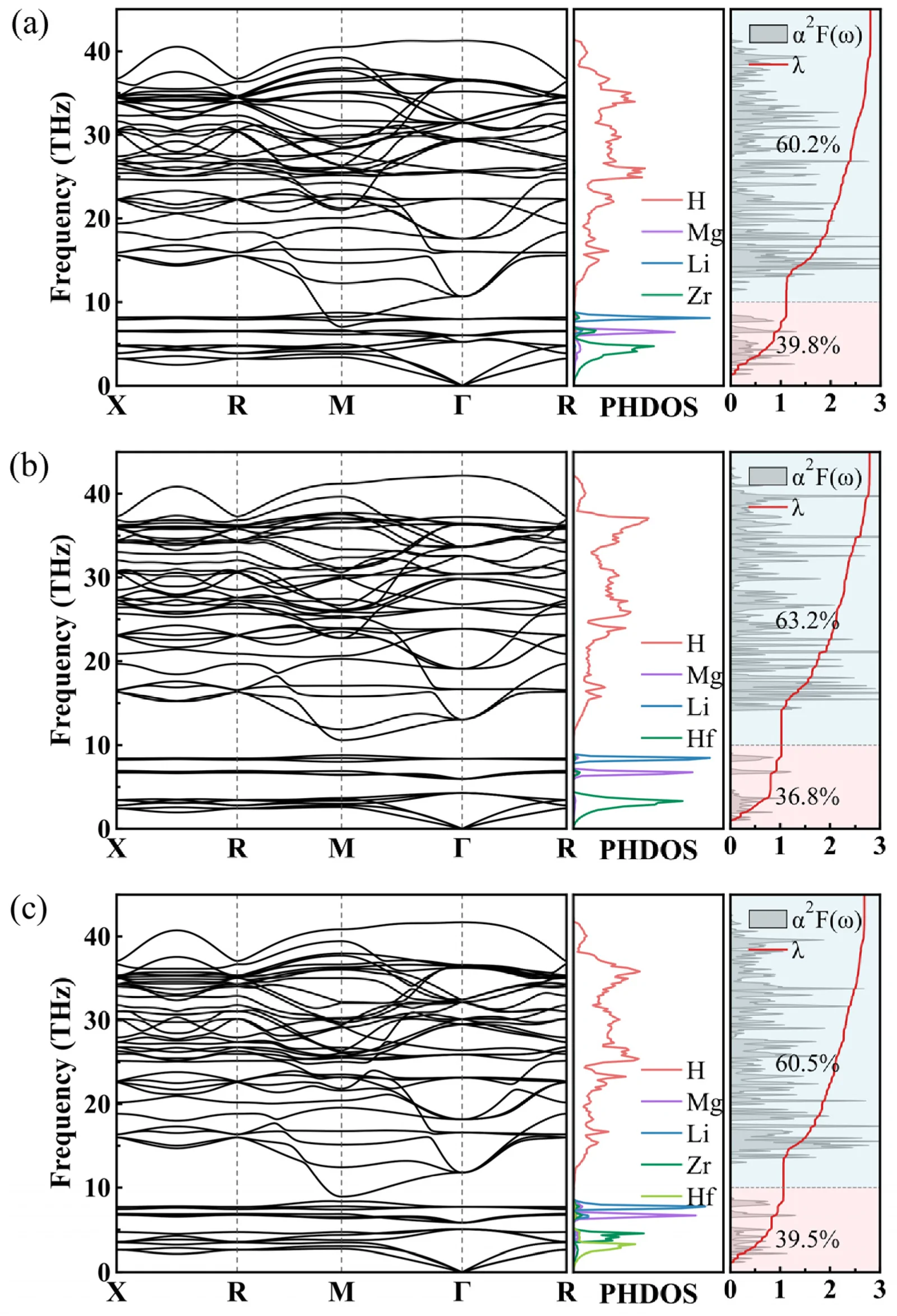
Calculated phonon dispersion curves, phonon density of states, Eliashberg phonon spectral function *α*^2^*F*(*ω*), and EPC parameter *λ* for (**a**) LiMgZr_2_H_12_, (**b**) LiMgHf_2_H_12_, and (**c**) LiMgZrHfH_12_.

Using the *Fm*-3-XYZ_2_H_12_ structure as the structural template, we constructed a series of LiMgM_2_H_12_ quaternary hydrides with *Fm*-3 symmetry, as shown in Figure 1a, where M represents 3*d*, 4*d*, and 5*d* transition metals (see Figure 1c). A total of 29 candidate compounds were generated for subsequent systematic screening. We first evaluated their mechanical stability according to the Born stability criteria [42] and excluded the structures that failed to satisfy these criteria. The dynamical stability of the remaining structures was then evaluated through phonon dispersion calculations. Structures exhibiting imaginary phonon frequencies were removed, and two dynamically stable structures at ambient pressure were finally obtained: LiMgZr_2_H_12_ and LiMgHf_2_H_12_. Based on these two compounds, we further constructed the quinary hydride LiMgZrHfH_12_ through atomic substitution. This compound was also found to be dynamically stable at ambient pressure. The phonon dispersion curves and crystal structural parameters of these three dynamically stable compounds are presented in Figure 2 and Table 1, respectively.

**Figure 3:**
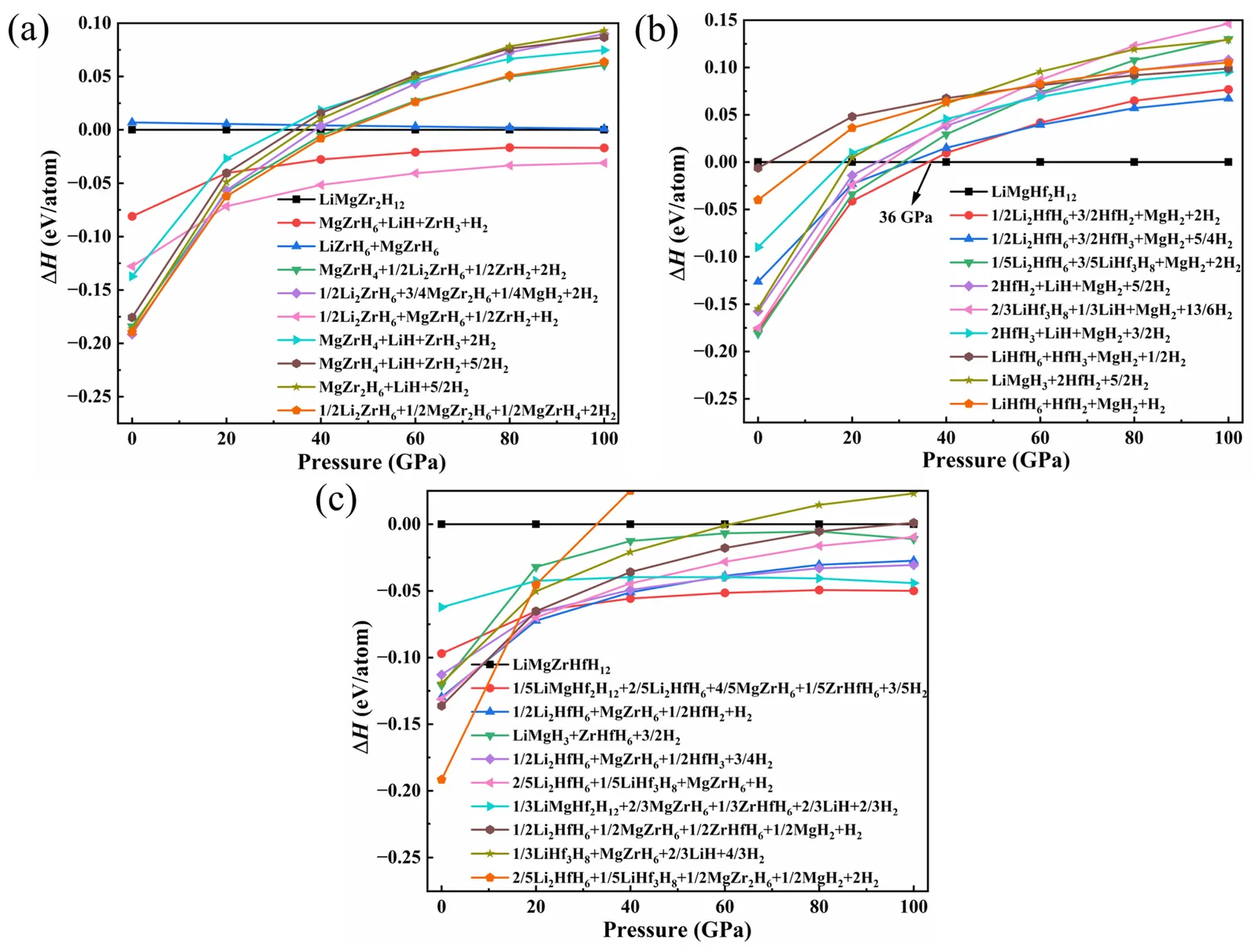
Relative enthalpies (Δ*H*) of (**a**) LiMgZr_2_H_12_, (**b**) LiMgHf_2_H_12_, and (**c**) LiMgZrHfH_12_ with respect to their corresponding decomposition products.

The thermodynamic stability of hydrides is an important criterion for evaluating their experimental synthesizability. To investigate the thermodynamic stability of LiMgZr_2_H_12_, LiMgHf_2_H_12_, and LiMgZrHfH_12_, we systematically compared their decomposition enthalpies with those of known binary, ternary, and quaternary competing phases. As shown in Figure 3, all three compounds are thermodynamically unstable at ambient pressure, whereas LiMgHf_2_H_12_ becomes thermodynamically stable above 36 GPa. The energies above the convex hull of these compounds are listed in Table 1, and all are below 0.2 eV atom^−1^, indicating their metastable nature. Notably, metastability does not necessarily preclude the experimental synthesizability of materials. Statistical analyses based on the Inorganic Crystal Structure Database (ICSD) have shown that approximately 20% of experimentally synthesized materials are metastable [43,44,45]. Recently, Deng et al. [46] developed a pressure-quench protocol and successfully retained the pressure-enhanced superconducting state in bulk tetragonal HgBa_2_Ca_2_Cu_3_O_8+δ_ after decompression to ambient pressure, achieving an ambient-pressure superconducting transition temperature of 151 K. Although cuprate superconductors differ substantially from the phonon-mediated hydrides investigated in this work in terms of their chemical bonding characteristics and microscopic pairing mechanisms, this study demonstrates that metastable superconducting phases can, in some cases, be retained at ambient pressure through pressure quenching. Based on this concept, pressure-assisted synthesis combined with controlled low-temperature decompression or pressure quenching may provide a potential route for the experimental preparation of the hydrides investigated herein. Nevertheless, this analogy offers only conceptual and methodological insights, and its practical feasibility remains to be further verified by future experimental studies.

**Figure 4:**
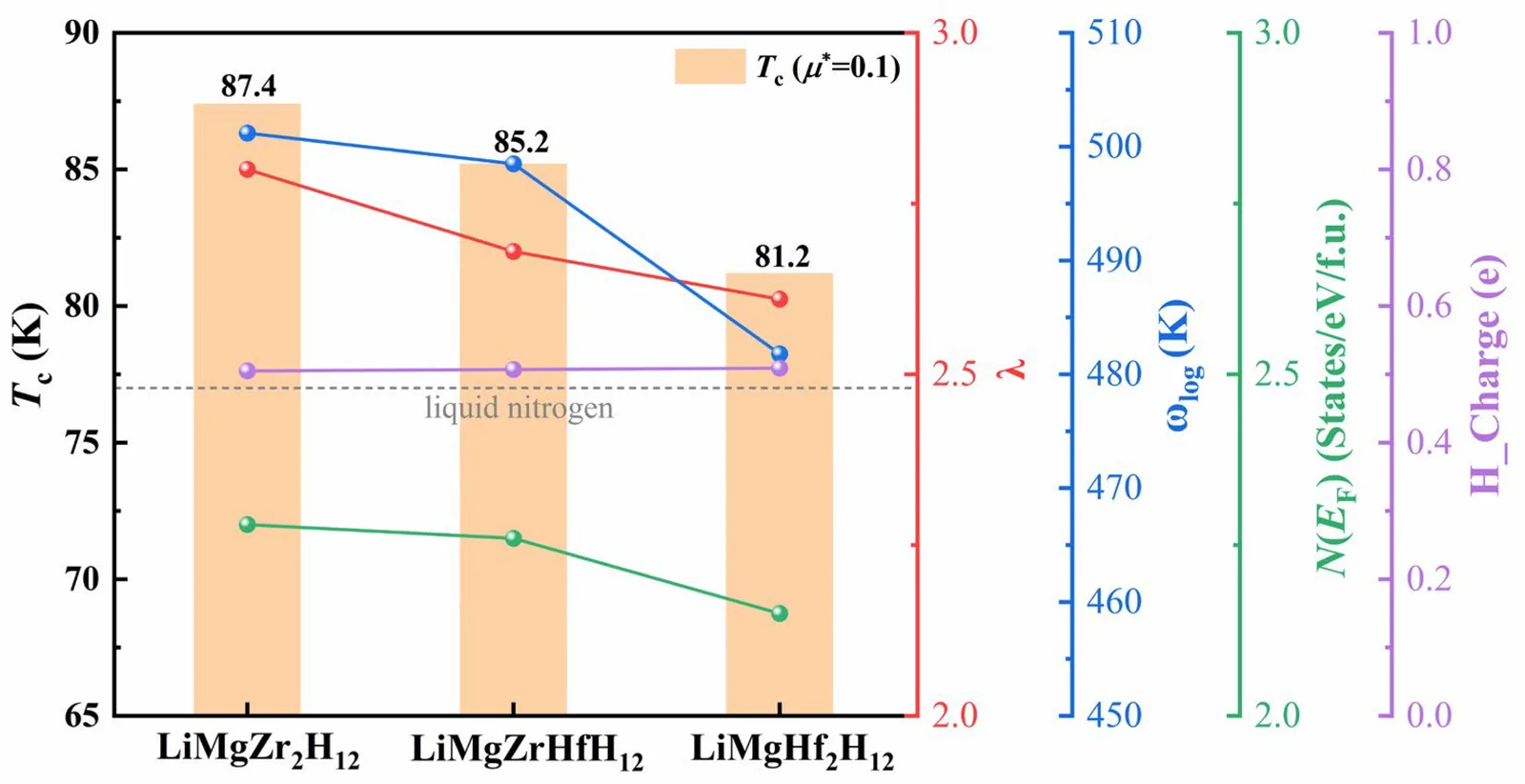
Superconducting parameters of LiMgZr_2_H_12_, LiMgHf_2_H_12_, and LiMgZrHfH_12_ at ambient pressure, including the superconducting transition temperature (*T*_c_) calculated using the Allen–Dynes modified McMillan equation (μ* = 0.1), the EPC parameter *λ*, the logarithmic average phonon frequency *ω*_log_, the electronic density of states at the Fermi level *N*(*E*_F_), and the average charge obtained by each H atom (H_Charge).

To investigate the superconducting properties of these three hydrides, their superconducting transition temperatures were determined by solving the Allen–Dynes-modified McMillan equation [34,47], and the corresponding results are listed in Table S1. For a Coulomb pseudopotential parameter μ* of 0.10–0.13, the ambient-pressure *T*_c_ values of LiMgZr_2_H_12_, LiMgHf_2_H_12_, and LiMgZrHfH_12_ are 82.9–87.4 K, 76.8–81.2 K, and 80.7–85.2 K, respectively. As shown in Figure 4, when μ* = 0.10, the *T*_c_ values of all three structures exceed the temperature of liquid nitrogen and are substantially higher than those of their parent compounds, GaMgZr_2_H_12_ (*T*_c_ = 20 K) and GaMgHf_2_H_12_ (*T*_c_ = 12 K) [25]. These results indicate that this class of Li–Mg-based multinary hydrides exhibits excellent superconducting properties. Furthermore, Figure 4 also summarizes the key superconducting parameters of these three hydrides. The calculated *T*_c_ values exhibit positive correlations with the electron–phonon coupling constant *λ*, the logarithmic average phonon frequency *ω*_log_, and the density of states at the Fermi level *N*(*E*_F_). Among the three compounds, the average electron gain of hydrogen atoms remains nearly unchanged (approximately 0.51 *e* per H atom), indicating that the substitution of Zr with Hf primarily modifies the metallic framework rather than directly altering the local bonding environment of hydrogen. In addition, the *λ* values of these three hydrides are all higher than 2.5, which are substantially larger than those of their parent structures, GaMgZr_2_H_12_ (*λ* = 0.66) and GaMgHf_2_H_12_ (*λ* = 0.57) [25]. This result indicates that the introduction of Li atoms markedly enhances the electron–phonon coupling strength of the system. Specifically, compared with Ga, Li has a lower electronegativity and can more readily transfer electrons to the hydrogen sublattice, thereby enhancing the ionic character of the structure and modifying the electronic filling of the hydrogen framework. This charge redistribution may promote the hybridization between the H-*s* states and the Zr/Hf-*d* states near the Fermi level, thereby contributing to the enhancement of electron–phonon coupling and the increase in the superconducting transition temperature. However, Li substitution not only induces charge redistribution but also modifies the lattice structure, local coordination, and chemical bonding environment. Therefore, the observed enhancement in superconducting properties is more likely attributable to the synergistic modulation of lattice structural evolution and electronic degrees of freedom.

To elucidate the microscopic mechanism underlying superconductivity in these hydrides, we calculated the phonon dispersion curves, projected phonon density of states (PHDOS), Eliashberg spectral function *α*^2^*F*(*ω*), and electron–phonon coupling constant *λ* for the three hydrogen-rich compounds, as shown in Figure 2. No imaginary phonon modes are observed throughout the Brillouin zone for any of the three compounds, confirming their good dynamical stability at ambient pressure. The EPC analysis shows that the phonon vibrations of LiMgZr_2_H_12_, LiMgHf_2_H_12_, and LiMgZrHfH_12_ can be divided into two distinct frequency regions: a low-frequency region dominated by metal atom vibrations and a high-frequency region dominated by hydrogen vibrations. Specifically, the vibrations of Li, Mg, and Zr/Hf atoms mainly contribute to the low-frequency phonon modes below 10 THz, whereas H vibrations dominate the high-frequency region above 10 THz. The Eliashberg spectral function *α*^2^*F*(*ω*) and electron–phonon coupling constant *λ* show that H-related phonon modes make the dominant contribution to the total EPC. In all three compounds, the contribution of high-frequency H vibrations to *λ* exceeds 60%. This indicates that the high-frequency vibrations of light H atoms not only provide high characteristic phonon frequencies but also play a key role in strengthening electron–phonon coupling.

**Figure 5:**
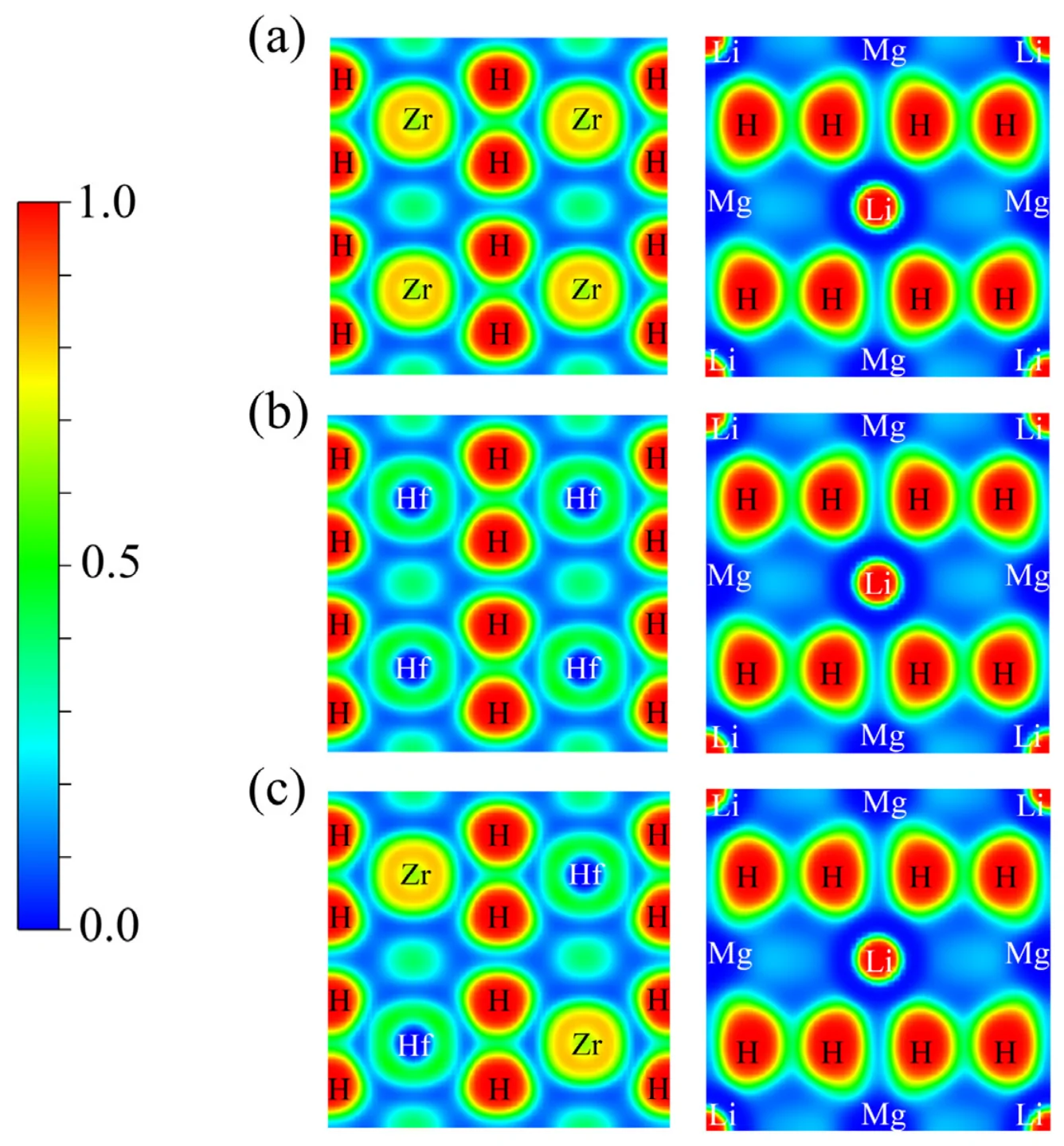
Electronic localization function (ELF) of (**a**) LiMgZr_2_H_12_, (**b**) LiMgHf_2_H_12_, and (**c**) LiMgZrHfH_12_.

To further elucidate the chemical bonding characteristics and charge transfer behavior of this series of hydrogen-rich compounds, we calculated the electronic localization function (ELF) [48] and performed Bader charge analysis for the three compounds. As shown in Figure 5, high ELF values are observed around the hydrogen atoms, whereas the regions between H and the metal atoms (Li, Mg, Zr, and Hf) exhibit low ELF values. This indicates pronounced charge transfer from the metal atoms to hydrogen, suggesting that the metal–H interactions in these systems are predominantly ionic. In addition, continuous electron localization is observed around neighboring H atoms, indicating that the hydrogen sublattice does not exist as isolated units but forms a relatively stable hydrogen-rich network. The ELF values between adjacent H atoms are all lower than 0.5, suggesting the absence of H–H covalent bonds and reflecting the anionic character of hydrogen. Further comparison of the ELF maps of these three structures reveals that their main differences arise from the transition metal–H interactions. Specifically, the Zr–H interaction is stronger than the Hf–H interaction, giving LiMgZr_2_H_12_ more pronounced metal–hydrogen hybridization characteristics. In contrast, LiMgHf_2_H_12_ exhibits relatively stronger ionic character, whereas LiMgZrHfH_12_ lies between the two and shows local bonding inhomogeneity caused by the different chemical environments of Zr and Hf. Such differentiated M–H bonding characteristics may modulate the electronic structure of the system and further influence its electron–phonon coupling behavior.

The Bader charge analysis further shows that Li, Mg, and Zr/Hf all act as electron donors, transferring electrons to the hydrogen framework, whereas the hydrogen atoms serve as electron acceptors, as summarized in Table S2. Specifically, the charge losses of Zr are 1.773 and 1.775 *e* in LiMgZr_2_H_12_ and LiMgZrHfH_12_, respectively. By comparison, Hf loses 1.800 and 1.802 *e* in LiMgHf_2_H_12_ and LiMgZrHfH_12_, respectively, indicating that Hf donates slightly more electrons to the hydrogen framework than Zr. This result is consistent with the ELF analysis, demonstrating that the Hf–H interaction is more ionic in nature, whereas the Zr–H region retains more pronounced electron localization and metal–hydrogen orbital hybridization. Notably, the average electron gain of H atoms in the three compounds is close to 0.51 *e*, exhibiting only weak compositional dependence. This indicates that the substitution of Zr by Hf mainly modulates the local electronic distribution of the metal framework, without significantly altering the overall electronic filling state of the hydrogen sublattice.

**Figure 6:**
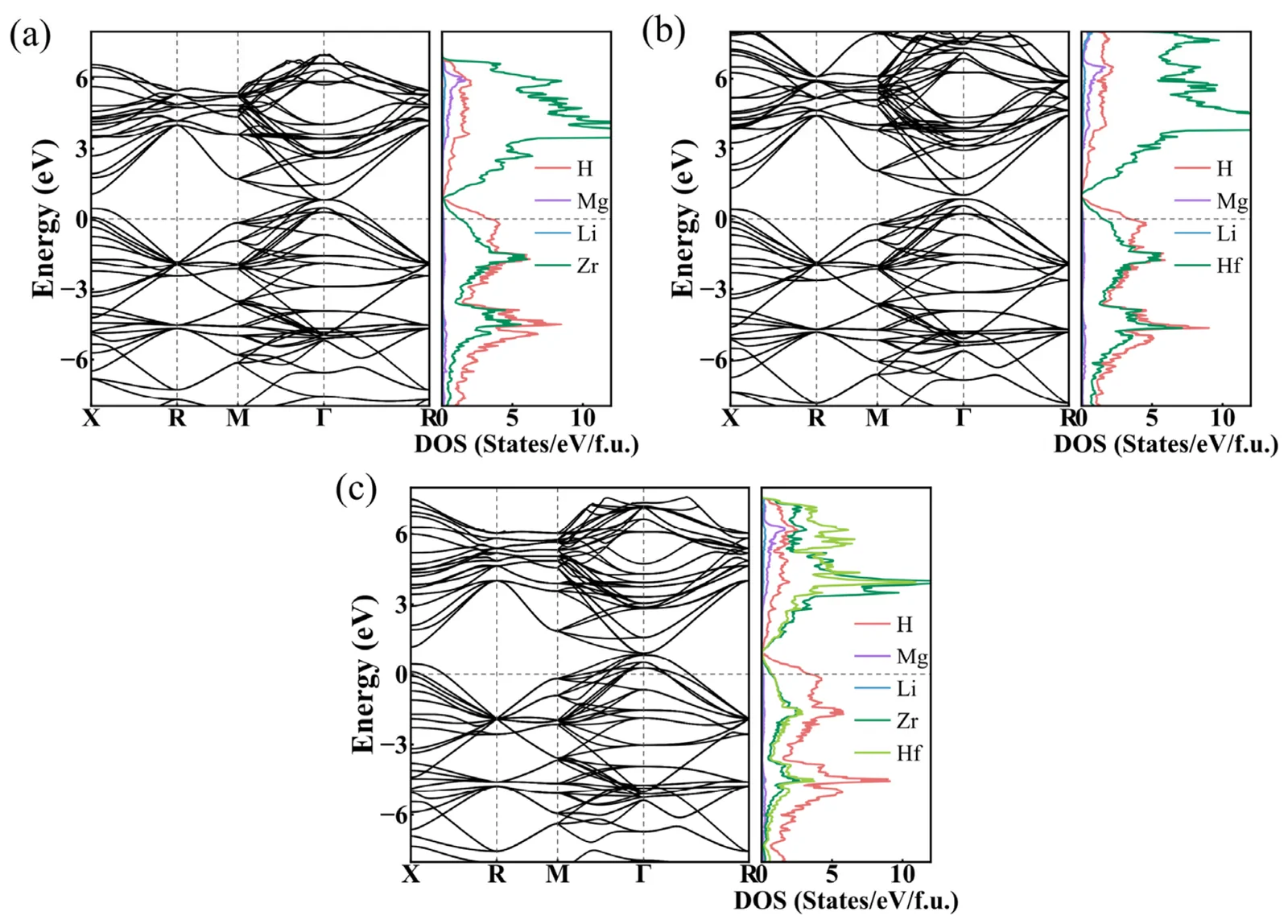
Calculated band structures and projected density of states (PDOS) of (**a**) LiMgZr_2_H_12_, (**b**) LiMgHf_2_H_12_, and (**c**) LiMgZrHfH_12_. The dashed line at zero denotes the Fermi energy.

Considering that superconducting properties are largely influenced by the distribution of electronic states near the Fermi level, we systematically analyzed the electronic band structures and density of states of the obtained structures to further clarify the effect of electronic structure on superconducting behavior. As shown in Figure 6, multiple bands cross the Fermi level, indicating that all three compounds exhibit metallic behavior. In LiMgZr_2_H_12_, LiMgHf_2_H_12_, and LiMgZrHfH_12_, H atoms make the most significant contribution to the density of states near the Fermi level. The electronic states near the Fermi level mainly originate from the hybridization between H-*s* states and Zr/Hf-*d* states. In contrast, Li and Mg make only minor direct contributions to the density of states near the Fermi level, indicating that Li and Mg atoms mainly act as electron donors and structural regulating components, rather than directly dominating the electronic states near the Fermi level that are associated with superconducting pairing. By comparison, the projected density of states (PDOS) of the parent *Fm*-3-XYZ_2_H_12_ structures reported in the literature shows that the electronic states near the Fermi level are mainly contributed by Zr/Hf-*d*, Ga-*s*, and H-*s* orbitals, with Zr or Hf atoms making the dominant contribution to the density of states near the Fermi level [25]. This difference indicates that the substitution of Ga by Li reconstructs the composition of electronic states near the Fermi level. As a result, the electronic states near the Fermi level change from being dominated by transition-metal *d* states in the parent structures to an electronic structure characterized by a dominant H-*s* contribution. According to the phonon-mediated BCS theory [49], H-dominated electronic states at the Fermi level are generally favorable for enhancing electron–phonon coupling and ultimately improving the superconducting transition temperature. This explains why the Li-based structures in this work exhibit superior superconducting performance compared with their Ga-based parent structures.

**Figure 7:**
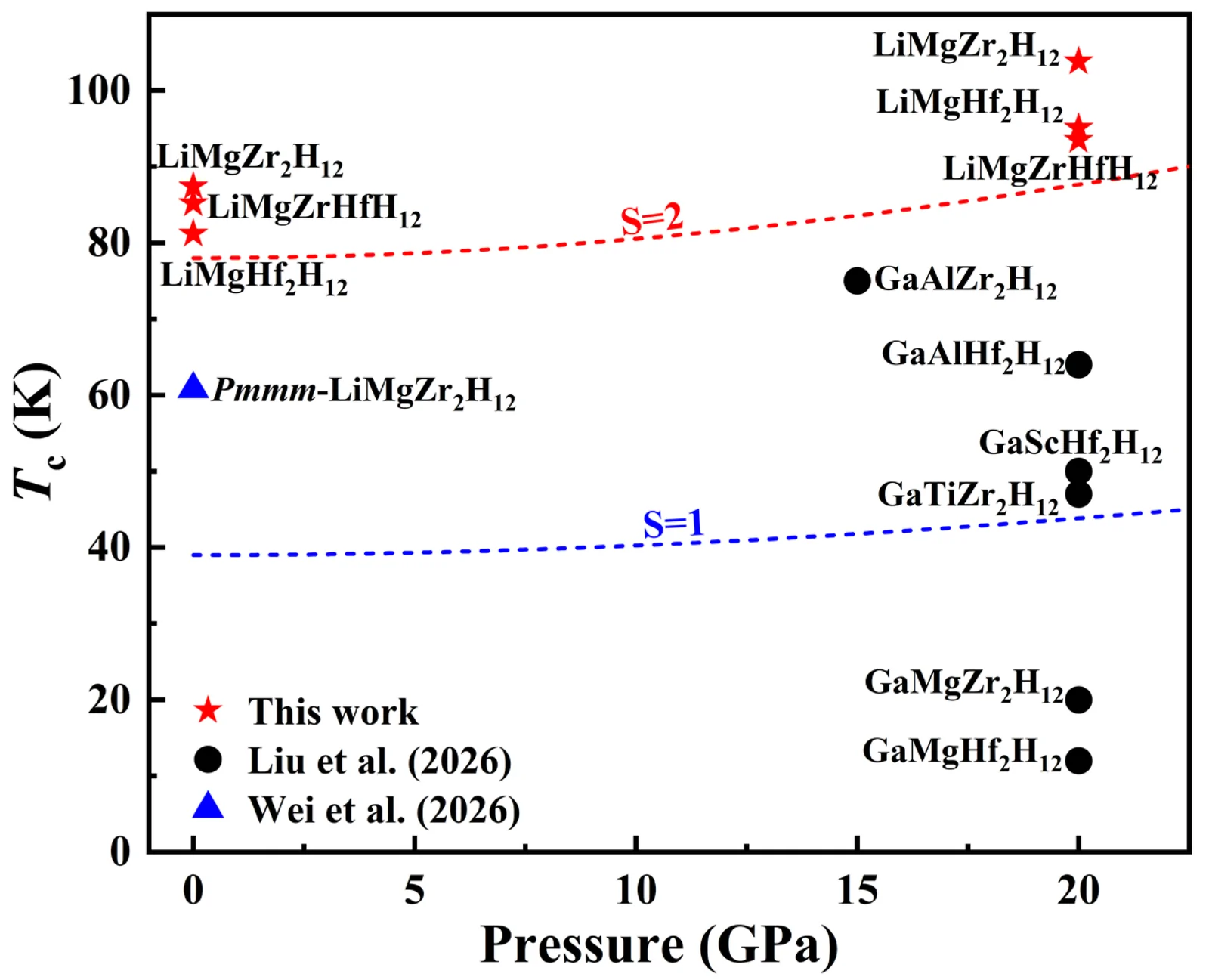
The superconducting transition temperature (*T*_c_) of LiMgZr_2_H_12_, LiMgHf_2_H_12_, LiMgZrHfH_12_, and representative quaternary hydride superconductors reported in the literature [25,26], calculated using the Allen–Dynes-modified McMillan equation with μ* = 0.10 at various pressures. The background dotted lines represent the superconducting figure of merit *S*.

To comprehensively evaluate the overall performance and potential application value of superconducting materials, Pickard et al. proposed a superconducting figure of merit *S* [50]. This metric simultaneously considers the transition temperature *T*_c_ of a material and the applied pressure required to achieve this temperature, thereby assessing the feasibility of superconducting systems under practical application conditions. *S* is defined as:

(7) $$S=T_{c}/\sqrt{T_{c,MgB_{2}}^{2}+P^{2}}$$

where $T_{c,MgB_{2}}$ represents the superconducting transition temperature for MgB_2_, and *P* denotes the applied pressure. To place the superconducting performance of the three hydrides investigated in this work into a broader context, Figure 7 compares them with several representative quaternary superconducting hydrides reported in the literature. As shown in Figure 7, the *S* values of LiMgZr_2_H_12_, LiMgHf_2_H_12_, and LiMgZrHfH_12_ all exceed 2. Among them, LiMgZr_2_H_12_ shows the most prominent performance, with an *S* value of 2.24. Notably, the *S* values of these three structures are not only significantly higher than those of their parent structures, GaMgZr_2_H_12_ (*S* = 0.46) and GaMgHf_2_H_12_ (*S* = 0.27), but also superior to those of other reported *Fm*-3-type XYZ_2_H_12_ compounds [25]. In addition, compared with the previously reported ambient-pressure superconducting phase *Pmmm*-LiMgZr_2_H_12_ [26], the *Fm*-3-type LiMgZr_2_H_12_ predicted in this work exhibits a higher superconducting transition temperature *T*_c_ at ambient pressure. Figure 7 further shows the variation in the superconducting transition temperatures of the three compounds under applied pressure. At μ* = 0.10, the *T*_c_ values of LiMgZr_2_H_12_, LiMgHf_2_H_12_, and LiMgZrHfH_12_ at 20 GPa are 103.8 K, 95.1 K, and 93.5 K, respectively. These values are significantly higher than the corresponding ambient-pressure values, indicating that pressure can further increase the theoretical superconducting transition temperatures of these compounds. Overall, the three hydrides proposed in this work exhibit high superconducting figures of merit, suggesting their considerable potential for practical applications.

## 4. Conclusions

Based on first-principles calculations, this study systematically investigates the structural stability and superconducting properties of quaternary LiMgM_2_H_12_ hydrides (M denotes a transition metal) and the quinary LiMgZrHfH_12_ hydride at ambient pressure. The results show that LiMgZr_2_H_12_, LiMgHf_2_H_12_, and LiMgZrHfH_12_ are dynamically stable at ambient pressure but thermodynamically metastable with respect to their competing decomposition products. Electron–phonon coupling calculations indicate that LiMgZr_2_H_12_, LiMgHf_2_H_12_, and LiMgZrHfH_12_ exhibit *T*_c_ values of 87.4, 81.2, and 85.2 K at ambient pressure, respectively. These values are all above the liquid-nitrogen temperature, demonstrating excellent superconducting performance. Further analysis shows that strong electron–phonon coupling is the key factor responsible for their excellent superconducting performance. Compared with the Ga-based parent *Fm*-3-XYZ_2_H_12_ hydrides, Li substitution at the Ga site synergistically modulates the local structure of the compounds and the distribution of electronic states near the Fermi level. This substitution significantly increases the contribution of H atoms to the density of states near the Fermi level, thereby enhancing the electron–phonon coupling and favoring an increase in the superconducting transition temperature. This work extends the elemental substitution strategy from quaternary to quinary hydride systems, not only enriching the existing library of ambient-pressure superconducting hydrides but also providing theoretical guidance for the experimental investigation of multicomponent hydride superconductors.

## Figures and Tables

**Table 1:** Optimized structural parameters and energies above the convex hull (*E*_hull_ in eV/atom) of LiMgZr_2_H_12_, LiMgHf_2_H_12_, and LiMgZrHfH_12_ at ambient pressure.

Structures	Lattice Parameter (Å)	Wyckoff Position				*E*_hull_
		Atoms	*x*	*y*	*z*	
LiMgZr_2_H_12_	*a* = *b* = *c* = 7.5631 *α* = *β* = *γ* = 90°	H (48*h*)	0.748	0.367	0.500	0.191
		Mg (4*b*)	0.500	0.500	0.500	
		Li (4*a*)	0	0	0	
		Zr (8*c*)	0.750	0.750	0.250	
LiMgHf_2_H_12_	*a* = *b* = *c* = 7.4929 *α* = *β* = *γ* = 90°	H (48*h*)	0.749	0.368	0.500	0.181
		Mg (4*b*)	0.500	0.500	0.500	
		Li (4*a*)	0	0	0	
		Hf (8*c*)	0.750	0.750	0.250	
LiMgZrHfH_12_	*a* = *b* = *c* = 7.5280 *α* = *β* = *γ* = 90°	H (48*h*)	0.249	0.867	0.498	0.192
		Mg (4*b*)	0	0	0.500	
		Li (4*a*)	0	0	0	
		Zr (4*c*)	0.250	0.750	0.750	
		Hf (4*d*)	0.250	0.250	0.750	

## Data Availability

The original contributions presented in this study are included in the article/Supplementary Material. Further inquiries can be directed to the corresponding authors.

## Supplementary Materials

The following supporting information can be downloaded at: https://www.mdpi.com/article/10.3390/ma19163465/s1, Figure S1: Phonon dispersion curves for (a) LiMgZr_2_H_12_, (b) LiMgHf_2_H_12_, and (c) LiMgZrHf_2_H_12_ at 20 GPa; Table S1: EPC constant *λ*, logarithmic average phonon frequency *ω*_log_ (in K), density of states at the Fermi level *N*_F_ (in States/eV/f.u.), and *T*_c_ values (in K) estimated using the Allen-Dynes modified McMillan equation with *μ** = 0.10–0.13 for LiMgZr_2_H_12_, LiMgHf_2_H_12_, and LiMgZrHf_2_H_12_ at selected pressures (in GPa); Table S2: The average charge value (*e*) and number of electrons transferred *δ* (*e*) for different atoms of LiMgZr_2_H_12_, LiMgHf_2_H_12_, and LiMgZrHfH_12_ at ambient pressure.
