# Supplementary material for: Designing Ambient Pressure Superconductivity in Li–Mg-Based Hydrides via Transition-Metal Modulation: From Quaternary to Quinary Systems
Source: Materials (Basel). 2026 Aug 16;19(16):3465. doi: 10.3390/ma19163465 (PMC13514449; doi:10.3390/ma19163465)
Supplement: Supplementary file 1 [file materials-19-03465-s001.zip › materials-4451566-supplementary.pdf]

Designing Ambient Pressure Superconductivity in Li–Mg-Based Hydrides via Transition-Metal Modulation: From Quaternary to Quinary Systems

Xinyu Wang[a], Qun Wei[a,*], Jing Luo[a], Meiguang Zhang[b,*]

[a] *School of Physics, Xidian University, Xi'an 710071, China*

[b] *College of Physics and Optoelectronic Technology, Baoji University of Arts and Sciences, Baoji 721016, China*

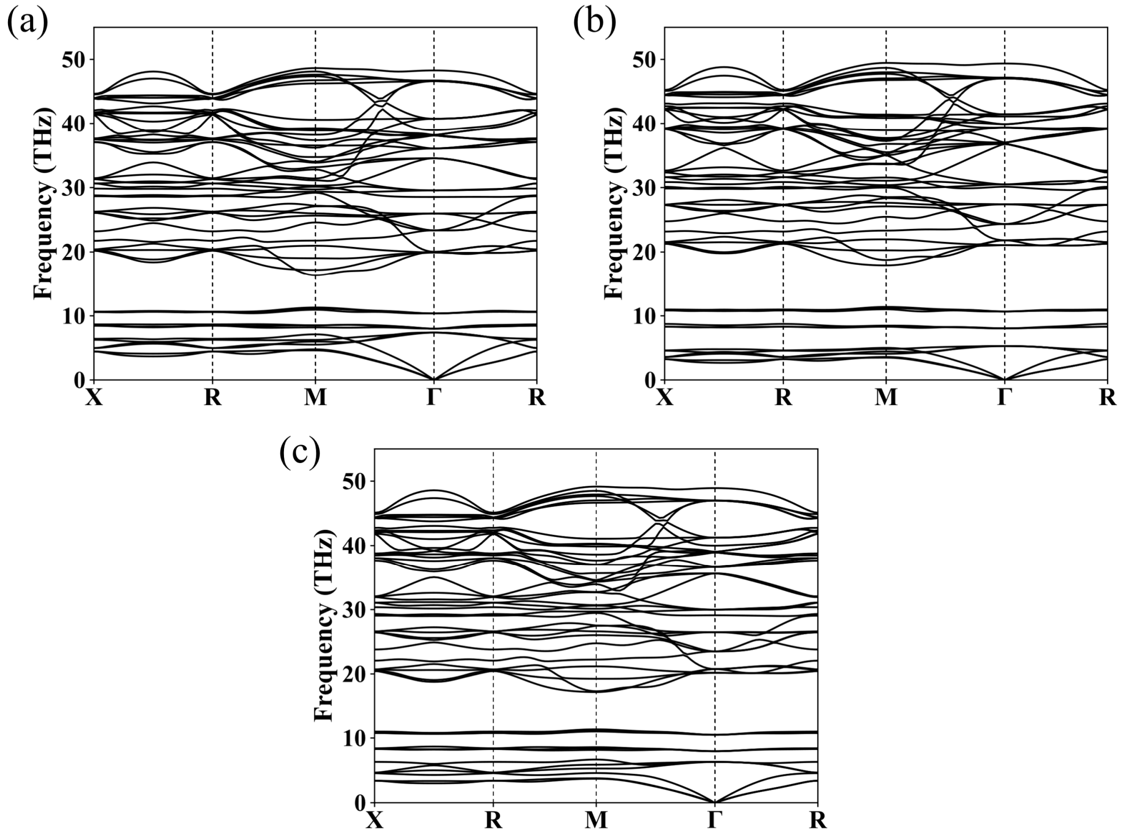

Figure S1. Phonon dispersion curves for (a) $LiMgZr_2H_{12}$, (b) $LiMgHf_2H_{12}$, and (c) $LiMgZrHf_2H_{12}$ at 20 GPa.

* Corresponding authors

E-mail address: qunwei@xidian.edu.cn (Q. Wei), mgbj@126.com (M. Zhang), and bwei@xidian.edu.cn (B. Wei)

Table S1. EPC constant λ, logarithmic average phonon frequency $\omega_{\log}$ (in K), density of states at the Fermi level N_F (in States/eV/f.u.), and T_c values (in K) estimated using the Allen–Dynes-modified McMillan equation with $\mu^* = 0.10–0.13$ for $LiMgZr_2H_{12}$, $LiMgHf_2H_{12}$, and $LiMgZrHf_2H_{12}$ at selected pressures (in GPa).

Structures	Pressure	λ	$\omega_{\log}$	N_F	T_c
$LiMgZr_2H_{12}$	0	2.80	501.2	2.28	82.9–87.4
	20	1.93	738.9	1.65	96.6–103.8
$LiMgHf_2H_{12}$	0	2.61	481.8	2.15	76.8–81.2
	20	1.86	695.3	1.74	88.3–95.1
$LiMgZrHfH_{12}$	0	2.68	498.5	2.26	80.7–85.2
	20	1.76	714.5	1.82	86.4–93.5

Table S2. The average charge value (e) and number of electrons transferred $\delta(e)$ for different atoms of $LiMgZr_2H_{12}$, $LiMgHf_2H_{12}$, and $LiMgZrHfH_{12}$ at ambient pressure.

Structures	Atom	Charge Value	$\delta(e)$
$LiMgZr_2H_{12}$	H	1.505	0.505
	Mg	0.365	-1.635
	Li	2.121	-0.879
	Zr	10.227	-1.773
$LiMgHf_2H_{12}$	H	1.509	0.509
	Mg	0.368	-1.632
	Li	2.123	-0.877
	Hf	8.200	-1.800
$LiMgZrHfH_{12}$	H	1.507	0.507
	Mg	0.367	-1.633
	Li	2.122	-0.878
	Zr	10.225	-1.775
	Hf	8.198	-1.802
